# Advances in the role of miRNAs in the occurrence and development of osteosarcoma

**DOI:** 10.1515/med-2020-0205

**Published:** 2020-10-12

**Authors:** Guanyu Zhang, Yiran Li, Jiasheng Xu, Zhenfang Xiong

**Affiliations:** Queen Mary college of Nanchang University, Xuefu Road, Nanchang, Jiangxi 330001, China; Department of Pathology, The First Affiliated Hospital of Nanchang University, Nanchang, China; Department of Pathology, The First Affiliated Hospital of Nanchang University, No. 17 Yongwaizheng Street, Nanchang, Jiangxi 330006, China

**Keywords:** osteosarcoma miRNA, diagnosis, prognosis

## Abstract

Osteosarcoma (OS) is the most common primary malignant tumor of the skeletal system in the clinic. It mainly occurs in adolescent patients and the pathogenesis of the disease is very complicated. The distant metastasis may occur in the early stage, and the prognosis is poor. MicroRNAs (miRNAs) are non-coding RNAs of about 18–25 nt in length that are involved in post-transcriptional regulation of genes. miRNAs can regulate target gene expression by promoting the degradation of target mRNAs or inhibiting the translation process, thereby the proliferation of OS cells can be inhibited and the apoptosis can be promoted; in this way, miRNAs can affect the metabolism of OS cells and can also participate in the occurrence, invasion, metastasis, and recurrence of OS. Some miRNAs have already been found to be closely related to the prognosis of patients with OS. Unlike other reviews, this review summarizes the miRNA molecules closely related to the development, diagnosis, prognosis, and treatment of OS in recent years. The expression and influence of miRNA molecule on OS were discussed in detail, and the related research progress was summarized to provide a new research direction for early diagnosis and treatment of OS.

## 1 Introduction

OS is one of the most common primary malignant tumors originating from mesenchymal tissue, mainly in children and adolescents, most commonly in the distal femur and proximal humerus. The current treatment is mainly surgery combined with neoadjuvant chemotherapy. However, surgery cannot effectively control the metastasis of tumors, and some patients are still not sensitive to chemotherapy. These problems, which led to the lung metastasis of tumors, have become major problems in the treatment of OS; so, improving the existing treatment concepts and looking for further new treatment technologies are imperative. miRNAs are endogenous single-stranded small RNAs of about 18–25 nt in length, which are involved in post-transcriptional regulation of genes, and have important effects on gene expression, biological development, and behavior [1,2]. Sayles et al. [4] found that the function of some miRNAs was determined by the phenotypic consequences of a mutated miRNA or an altered miRNA complementary site, either of which can disrupt miRNA regulation. In other cases, function was inferred from the effects of mutations or transgenic constructs that lead to miRNA ectopic expression. Existing studies have shown that there are significant changes in miRNA expression profiles in a variety of human benign or malignant diseases, and the abnormal expression of miRNAs has an important relationship with the occurrence and development of diseases [2,3]. Each miRNA has a specific target mRNA, and the mRNA encoded by the tumor suppressor or tumor-promoting gene can affect the synthesis of some important cancer-associated functional proteins, so changes in mRNA expression can directly affect the occurrence of malignant tumors [4]. At the same time, this effect is very complex, because each miRNA has a large number of regulatory targets (some of which can reach hundreds of targets), and even small changes in expression may have a significant impact on the cell function, which in turn affects malignancy. A large number of studies have also shown that there are significant changes in the miRNA expression profile of OS patients, and miRNAs participate in the occurrence, development, and invasion of OS through various mechanisms. At the same time, a large number of clinical and basic research studies have also explored the value of miRNA for the diagnosis, prognosis, and treatment of OS patients. Some miRNAs have already been found to be closely related to the prognosis of patients with osteosarcoma (OS). Unlike other reviews, we briefly reviewed the research progress of the functional role of miRNAs in OS in recent years in this review, in order to provide new ideas for the clinical treatment of OS.

## 2 miRNA and OS occurrence and development

**Figure 1:**
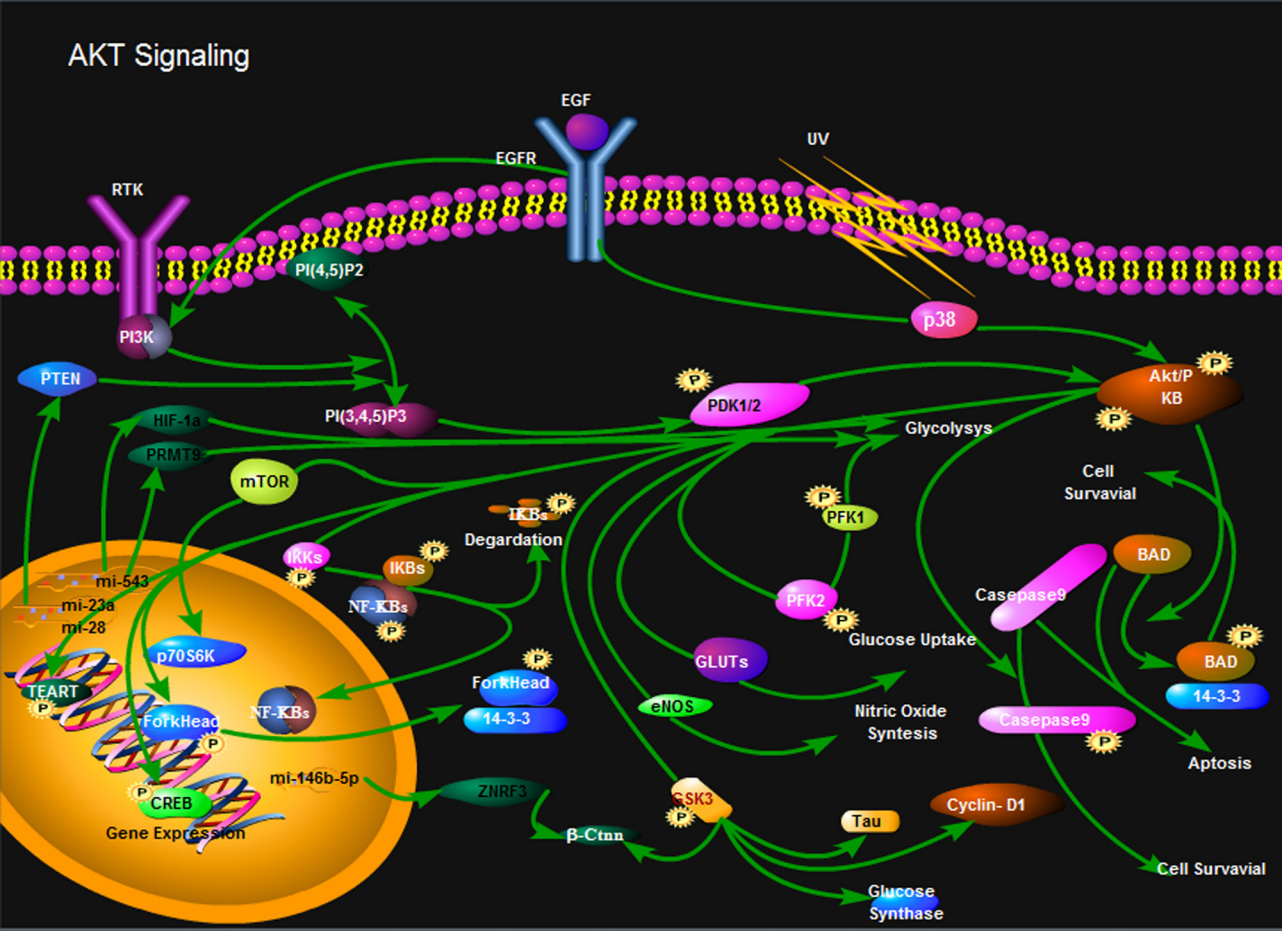
miRNA-23a can enhance the metastasis and invasion of OS cells by upregulating the signal expression of PTEN signaling pathway.

The occurrence and development of OS is affected by many factors including genetics, inflammation, and environment. Other factors include age, gender, ethnicity, and preexisting bone abnormalities. It is a very complicated pathophysiological process. Changes in miRNA expression profiles are involved in the development and progression of OS through a variety of complex direct or indirect regulatory actions. Some miRNAs promote the proliferation and differentiation of OS cells by significantly upregulating expression in OS [3]. The study found that the miRNA-128 expression level was significantly increased in OS tissues compared with normal skeletal tissues adjacent to the cancer, and miRNA-128 was mainly achieved by directly regulating the PTEN/AKT signaling pathway (involved in the protein synthesis of tumor cells). At the same time, studies have shown that miRNA-23a is similar to miRNA-128 and can also enhance the metastasis and invasion of OS cells by upregulating the signal expression of PTEN signaling pathway (Figure 1) [5]. Zhang et al. [6] found that miRNA-543 can partially inhibit the function of PRMT9 protein and stabilize HIF-1α protein, thereby promoting the proliferation and glycolysis of OS cells and promoting the progression of OS. Studies by Xu et al. [7] have also shown that miRNA-146b-5p promotes OS cell invasion and metastasis in patients with chemotherapy-resistant OS. The mechanism is mainly through miRNA-146b-5p against zinc finger protein-3 (ZNRF3). Direct targeting is achieved. In addition, studies have shown that miRNA-374a can promote the proliferation of OS cells by targeting the expression of AXIN2 functional proteins associated with malignant transformation [8]. miRNA-130b can target NKD2 to regulate the expression of WNT signaling pathway, thereby promoting the proliferation of OS cells and inhibiting autophagy of OS cells, and promoting the progression of OS [9].

There are also significant expression reductions in various miRNAs in OS, which may regulate the development and progression of OS through some important negative regulatory mechanisms. Such as Sun et al. [10] added diallyl trisulfide to the OS cell line cultured in vitro and revealed that the expression of miRNA-34a, miRNA-143, miRNA-145, and miRNA-200b/c was absent in OS cells with inhibited growth and invasion ability. And this loss of expression is associated with inhibition of the Notch signaling pathway.

Novello et al. [11] have found that there is a decrease in the expression of the entire miRNA-34 family in OS tissues compared to normal skeletal tissues. The related mechanism may be that miRNA-34a can directly affect the transcription of P53 gene and downregulate c-MET gene to inhibit the proliferation and metastasis of OS cells. Therefore, the decrease of miRNA-34a expression can promote the occurrence and development of OS. At the same time, studies have shown that the miRNA-34 family (miRNA-34a, miRNA-34b, and miRNA-34c) can also affect multiple target genes including the Notch signaling pathway, thereby affecting the pathogenesis of OS [12]. In addition, Zhang et al. [13] studies have also shown that miRNA-34c can not only play a carcinogenic role by inhibiting the expression of the Notch signaling pathway, but also directly inhibit the normal differentiation of osteoblasts, leading to a cell cycle regulation disorder. Other reduced-expressing miRNAs, such as miRNA-218, were also reduced significantly in human OS tissues and OS cell lines (SAOS-2 cells), and transfection of miRNA-218 gives SAOS-2 cells the ability to invade and metabolize distantly. The ability has also been significantly suppressed and the related mechanism is that the three major regulatory target genes are metastasis factor 1, matrix metalloproteinases (MMP)-2 and MMP-9, and the proteins encoded by these three genes are also important functional proteins involved in the invasion and metastasis of OS [14].

## 3 Diagnosis and prognosis of miRNA and OS

At present, the clinical diagnosis of OS mainly depends on the imaging examination and tissue biopsy. However, with the in-depth study of miRNA function mechanism in OS, the change of miRNA in blood circulation can also be used as a non-invasive auxiliary index for diagnosis and prognosis of OS. After the wide application of clinical chemotherapy, the long-term survival rate of OS patients has been significantly improved [15,16]. Tumor response to chemotherapy is one of the most important prognostic factors for OS, and those with a good response to chemotherapy may have a 10-year survival rate of 73%. How to apply the differential expression of miRNA to clinical diagnosis and prognosis is also the focus of current scholars. Unfortunately, even though chemotherapy has been greatly improved, a significant proportion of patients have a poor response to treatment, with a higher risk of recurrence or metastasis after extensive resection and chemotherapy. There are clinical or imaging metastases in 20–25% of OS patients. In patients who are determined to have metastases, the 5-year survival rate is only about 30%. Moreover, there are currently no effective biomarkers in the clinic that can be used to identify patients with a strong tumor aggressiveness and to screen patients who respond well to chemotherapy regimens.

An area under the curve (AUC) of 0.8606 for diagnosis of OS and a significant reduction in miRNA-199a-5p after OS surgery (Table 1) can be used as indicators of efficacy evaluation. Wang et al. [17] made a study of 80 patients with OS and showed that serum miRNA-664 alone diagnosed OS with an AUC of 0.956, a specificity of 92.5%, and a sensitivity of 96.2%. Wang et al. [18] have found that serum miRNA-191 levels are used to differentiate between OS and healthy patients with an AUC of up to 0.8 08, high serum miRNA-191 levels and clinical stage (*P* = 0.001), tumor volume (*P* = 0.01), and distant metastasis (*P* = 0.001) showed a significant positive correlation, and multivariate analysis showed that serum high miRNA-191 levels were independent risk factors for short-term mortality (*P* = 0.01). de Azevedo et al. [19] investigated the diagnostic and prognostic value of miRNA-221 expression levels in patients with OS. There was a significant positive correlation between high miRNA-221 levels and distant metastasis (*P* = 0.01) and high tumor clinical stage (*P* = 0.006). The AUC for the diagnosis of OS was 0.844, and patients with high miRNA-221 levels had a worse prognosis. Another study included 166 patients with OS. The results showed that miRNA-27a differentially diagnosed OS and healthy controls with an AUC of 0.867, and high miRNA-27a expression levels with higher clinical stage (*P* = 0.001), distant metastasis (*P* = 0.01), and poor response to chemotherapy (*P* = 0.008) [20]. Liu et al. [21] developed a study of OS patients (*n* = 114), where the serum miRNA-300 levels in OS patients were significantly higher than those in healthy patients, and the AUC for diagnosis of OS was 0.884, and multivariate regression analysis showed that miRNA-300 (HR = 5.964, *P* = 0.009), clinical stage (HR = 4.936, *P* = 0.011), and distant metastasis (HR = 6.102, *P* = 0.006) were independent prognostic factors affecting the overall survival of OS patients. In addition, such as studies by Lian et al. [22] have shown that patients with an increased miRNA-1908 expression have higher recurrence, metastasis, and poor response to chemotherapy. The overall survival rate of OS with low miRNA-1908 expression is higher, suggesting that miRNA-1908 can be a poor prognosis index for OS. Studies by Wang et al. [23] have shown that the presence of miRNA-17-5p is elevated in cancer tissues of OS patients and can be used as an indicator of diagnosis and prognosis in OS patients, and that miRNA-17-5p promotes OS proliferation mainly through BRCC2-dependent signaling mechanisms.

**Table 1:** miR-199a-5p Ct of control and pre-operative OS

miRNA	Ct of healthy control	Ct of pre-operative OS	ΔCt (pre-operative−control)
miR-199a-5p	34.35248	26.51492	−7.83756

On the other hand, the reduction of miRNA expression in some OS patients can also be used as a negative judgment indicator for diagnosis and prognosis. Tang et al. [24] studied 18 patients with OS before surgery and 50 patients after surgery, and four serum miRNAs (miRNA-195-5p, miRNA-199a-3p, miRNA-320a, and miRNA-374a-5p) were analyzed for OS diagnosis. The results showed that the efficacy of diagnostic AUC reached 0.9608 (95% CI: 0.9300–0.9912), and these four miRNAs showed a significant decline after surgery, and miRNA-195-5p and miRNA-199a-3p are associated with the presence of distant metastasis, whereas miRNA-320a is associated with biopsy staging. Such as Liu et al. [25] developed a study of 122 patients, miRNA-126 expression levels in OS tissues were significantly lower than in adjacent normal skeletal tissues (2.421 ± 1.250 vs. 6.12 ± 1.843, *P* = 0.001), and miRNA-126 was associated with higher TNM staging (*P* < 0.001), and Kaplan–Meier analysis showed that patients with a lower miRNA-126 expression had a shorter overall survival time (*P* = 0.008) (Table 2).

**Table 2:** Summary of important miRNA type associated with OS

miRNA type	Relative level in OS tissues	Pathway involved	Effect of miRNA
miRNA-128	Increased	PTEN/AKT signaling pathway	Enhances the metastasis and invasion of OS cells
miRNA-23a	Increased	PTEN signaling pathway	Enhances the metastasis and invasion of OS cells
miRNA-543	Increased		Inhibits the function of PRMT9 protein, stabilizes HIF-1α protein, and promotes the proliferation and glycolysis of OS cells
miRNA-146b-5p	Increased		Promotes OS cell invasion and metastasis by ZNRF3
miRNA-374a	Increased		Promotes the proliferation of OS cells by targeting the expression of AXIN2 functional proteins
miRNA-34a	Reduced	Notch signaling pathway	Affects the transcription of P53 gene and downregulates c-MET gene to inhibit the proliferation and metastasis of OS cells
miRNA-143, miRNA-145, and miRNA-200b/c	Reduced	Notch signaling pathway	Inhibit the proliferation and metastasis of OS cells
miRNA-34c	Reduced	Notch signaling pathway	Directly inhibits the normal differentiation of osteoblasts, leading to a cell cycle regulation disorder
miRNA-191	Increased		Risk factors for short-term mortality and related to distant metastasis
miRNA-221	Increased		Related to distant metastasis and high tumor clinical stage
miRNA-27a	Increased		Related to higher clinical stage, distant metastasis (*P* = 0.01), and poor response to chemotherapy
miRNA-17-5p	Increased	BRCC2-dependent signaling pathway	Promotes OS proliferation
miRNA-126	Reduced		Associated with higher TNM staging, shorter overall survival time
miRNA-106b	Reduced	PI3K/AKT signaling pathway	Regulates cell cycle G1/S transformation and regulates the invasion ability of U2OS OS cells
miR-223	Reduced	JNK signaling pathway	Negatively regulates the expression of Hsp70 in OS cells treated with cisplatin and produces drug resistance of cisplatin
miRNA-218	Reduced	Three major regulatory target genes are metastasis factor 1, matrix met alloproteinases (MMP)-2 and MMP-9	Involved in the invasion and metastasis of OS
miRNA-199a-5p	Increased		Serves as an indicator of efficacy evaluation
miRNA-300	Increased		Shows higher recurrence, metastasis, and poor response to chemotherapy, poor prognosis
miRNA-1908	Increased		Shows prognostic factors affecting the overall survival of OS patients
miRNA-195-5p and miRNA-199a-3p	Increased		Offers distant metastasis
miRNA-320a	Increased		Associated with biopsy staging
miR-150	Reduced		Acts as an anti-cancer regulator and acts on ZEB1 to inhibit the development of OS
miR-208b	Reduced		Downregulates the expression of ROR2 (receptor tyrosine kinase-like orphan receptor 2) gene, it can be used as a new target to prevent tumor metastasis
miR-210	Increased		Involved in hypoxia and promotes the differentiation of OS cells by activating TGF-β1 and its downstream factors, thereby promoting the tumor development and metastasis
miR-27-3p	Increased		Inhibits the anti-cancer effect of ING5 and promotes the proliferation of OS cells
miR-133b	Increased		Enhanced drug resistance of tumor cells to cisplatin, it can be used as a biomarker for detecting whether or not drug resistance is produced
miR-340	Decreased		Increases drug resistance behavior in OS, it can be used as a biomarker to alleviate the drug resistance of OS
miR-25-3p	Increased		Used as an important non-invasive biomarker for patient OS monitoring and prognosis assessment
miR-199a-3p	Decreased		Increases the growth as well as the development of OS

## 4 miRNA and OS treatment

In recent years, many studies have shown that miRNA molecules not only participate in the regulation of OS growth microenvironment, tumor resistance, and tumor cell exocytosis, but can also be used to prepare nanoparticles and target OS. The analysis of the mechanism of action of miRNA molecules in the above events will help provide new ways and means for the clinical treatment of OS. The following are introduced to participate in the regulation of OS growth microenvironment, tumor resistance, tumor cell exocytosis function, and miRNA molecules for nanotargeted therapy and their functional characteristics.

### 4.1 miRNA molecules that regulate the growth of OS microenvironment

Many miRNAs are upregulated or downregulated in different types of tumor microenvironments, effectively regulating the expression of these miRNAs and inhibiting tumor growth. Xu et al. [26] found that miR-150 expression was downregulated in OS tissues and cells, followed by the transfection of OS cells, using ZEB1 (zinc finger E-box binding homeobox 1) siRNA technology, MTT assay, and Transwell assay using miR-150 mimics. It was found that miR-150 acts as an anti-cancer regulator and acts on ZEB1 to inhibit the development of OS. Jiang et al. [27] found that miR-208b was downregulated in OS cells and then found that miR-208b downregulated the expression of ROR2 (receptor tyrosine kinase-like orphan receptor 2) gene by cell transfection, PCR, and WB. Transfection of ROR2-siRNA into OS cells revealed that ROR2 silencing inhibits the proliferation, invasion, and migration of OS cells, so miR-208b can be used as a new target to prevent tumor metastasis. Zhang et al. [28] found that miR-210 significantly increased expression during dedifferentiation of OS cells. After upregulation of miR-210 expression, it is involved in hypoxia and TGF-β1-induced OS cell differentiation. In MNNG/HOS cells, miR-210 is positively correlated with TGF-β1 expression, and miR-210 can promote the differentiation of OS cells by activating TGF-β1 and its downstream factors, thereby promoting the tumor development and metastasis. Further data suggest that NFIC (nuclear factor I/C) is a target gene for miR-210 in MNNG/HOS cells, and miR-210 expression is negatively correlated with NFIC expression, suggesting that NFIC may be a potential target gene involved in the differentiation of OS cells. Xu et al. [29] found that due to the inhibition of miR-106b expression, OS cell proliferation rate was significantly reduced, and the WB detection analysis showed that the phosphorylation level of the key protein AKT in the PI3K/AKT signaling pathway and the expression level of PI3K catalytic subunit were significantly decreased, indicating that miR-106b can promote the activation of PI3K/AKT signaling pathway in OS cells, regulate cell cycle G1/S transformation, and regulate the invasion ability of U2OS OS cells.

Ye et al. [30] observed the upregulation of miR-27-3p expression in OS cells. Using OS cells such as U2OS and MG63, it was found that exogenous miR-27-3p overexpression can directly target the tumor growth inhibitor ING5. In addition, it inhibits the anti-cancer effect of ING5 and promotes the proliferation of OS cells. The above studies show that miRNA can regulate the microenvironment of OS cell proliferation, invasion, and migration through different regulation methods, and inhibit the growth and development of OS.

### 4.2 Targeting miRNA molecules related to OS drug resistance

Long-term use of certain chemotherapeutic drugs in patients with OS is easy to induce gene mutations, resulting in drug resistance, and thereby increasing the difficulty of treatment of OS. In response to this problem, many scholars have studied the mechanism of OS resistance and the key miRNA molecules involved in the regulation of drug resistance. Zou et al. [31] found that the expression level of miR-133b in cisplatin-resistant OS MG63 cells (MG63-DDP) was elevated. The miR-133b precursor (pre-miR-133b) was transfected into the cell, and the expression of miR-133b was upregulated and resistant to cisplatin. It is speculated that the expression of miR-133b is increased in patients with OS after long-term cisplatin chemotherapy, resulting in an enhanced drug resistance of tumor cells to cisplatin. Therefore, miR-133b can be used as a biomarker for detecting whether or not drug resistance is produced. Zou et al. [32] found that the miR-340 expression was downregulated in OS cells and that the ZEB1 expression was upregulated. Further studies revealed that miR-340 and ZEB1 expression levels were associated with drug resistance. Transfection of miR-340 by MG63-DDP cells upregulated miR-340 expression, acting on the ZEB1 gene, thereby reducing drug resistance behavior in OS. Therefore, miR-340 can be used as a biomarker to alleviate the drug resistance of OS. Mujtaba et al. [33] examined the expression levels of miR-223 in 20 pairs of OS and non-tumor tissues, and found that the miR-223 expression was significantly downregulated in OS tissues. When MG63 cells were transfected with miR-223 mimics, miR-223 expression was upregulated and the cell proliferation rate was slowed down. miR-223 can negatively regulate the expression of Hsp70 in OS cells treated with cisplatin and produce drug resistance of cisplatin through the JNK signaling pathway.

In summary, miRNA molecules are involved in the drug resistance behavior of OS. They can not only be used as a novel biomarker for judging whether or not OS produces drug resistance, but can also regulate the drug resistance behavior of OS and become an important treatment target for OS [34,35,36,38].

### 4.3 Extracorporeal function of miRNA molecules related to tumor cells

miRNAs in OS exosomes can be released into body fluids (e.g., blood, urine, etc.), which can serve as potential biomarkers for disease. Fujiwara et al. [39] collected 14 OS patients, 14 age-related non-OS patients, and eight normal human serum patients, and compared the miR-25-3p in serum and OS cells of the above three different populations. The expression level of miR-17-5p was changed. It was found that the OS cells secreted miR-25-3p and miR-17-5p, and the expression level of serum miR-25-3p in OS patients was significantly higher than those of the other two groups. The specificity and sensitivity of this indicator (serum miR-25-3p) (71.4% and 92.3%, respectively) are superior to those of traditional alkaline phosphatase assays, making serum miR-25-3p to be used as an important non-invasive biomarker for patient OS monitoring and prognosis assessment. Unfortunately, due to the limited resources of clinical samples, it is rarely used in the early treatment of clinical OS. It is believed that the clinical research of large samples in the future will provide theoretical support for its clinical treatment. Therefore, exosome-associated miRNA molecules have potential applications in the early diagnosis and treatment of OS [10,40,41].

### 4.4 Nanotargeted therapeutic-related miRNA molecules

With the rapid development of nanotechnology, the application of nanoparticles to carry miRNAs, through the nanosize effect (EPR effect), has become possible to achieve targeted therapy of tumors. Zhang et al. [42] found that miR-199a-3p expression was decreased in OS cells, and upregulation of its expression inhibited the growth of OS. Therefore, they designed and synthesized a lipid-modified dextran-based polymer nanoparticle system to encapsulate miR-199a-3p molecules, to achieve targeted delivery to tumors, and to inhibit the growth as well as the development of OS. The miRNA-let-7a with tumor suppressor effect was used as a positive control. It was found by fluorescence microscopy and real-time PCR that dextran-based polymer nanoparticles can deliver miR-199a-3p and let-7a to KHOS and U2OS. In OS cells, further studies have found that polymer nanoparticles loaded with miR-199a-3p can effectively downregulate the expression of related target proteins, thereby inhibiting the growth of OS cells. This study demonstrates that lipid-modified dextran-based polymer nanoparticles are effective non-viral vectors that can be used to deliver miRNA molecules with an enhanced targeting potential for miRNAs. Therefore, in combination with advanced nanopreparation techniques and the functional characteristics of miRNA molecules themselves, it is not only possible but also feasible to apply miRNA-coated nanoparticles to OS.

## 5 Conclusion

In summary, miRNAs play an important role in the development, invasion, metastasis, and prognosis of OS. The differential expression of miRNAs in OS cells provides a new target for the early diagnosis and treatment of OS. The study of drugs affects the expression level of miRNAs in OS cells, which in turn affects the progression and prognosis of tumors, and provides a new direction for the study of molecular targeted drugs for the treatment of OS [43–46]. However, although there is a deep understanding of the differential expression and functional roles of some miRNAs in OS, there are still many difficulties to overcome in order to treat OS through the targeting of miRNAs: the sequence error of the miRNA sequence library, poor RNA extraction methods, variability in detection and analysis, diversity of bioinformatics analysis, and non-standardization of miRNA clinical tests [47,48]. In conclusion, the exploration of the relationship between miRNAs and OS provides new ideas and challenges for the early diagnosis, molecular targeted therapy, and prognosis of OS [49–51]. It is believed that with the deep understanding of the mechanism of action of miRNA molecules in important events such as the occurrence, proliferation, invasion, and metastasis of OS, miRNA is expected to be applied to the clinical precision treatment and prognosis evaluation of OS in the near future.
